# Demonstrating quality control procedures for fMRI in DPABI

**DOI:** 10.3389/fnins.2023.1069639

**Published:** 2023-02-21

**Authors:** Bin Lu, Chao-Gan Yan

**Affiliations:** ^1^CAS Key Laboratory of Behavioral Science, Institute of Psychology, Beijing, China; ^2^Department of Psychology, University of Chinese Academy of Sciences, Beijing, China; ^3^International Big-Data Center for Depression Research, Institute of Psychology, Chinese Academy of Sciences, Beijing, China; ^4^Magnetic Resonance Imaging Research Center, Institute of Psychology, Chinese Academy of Sciences, Beijing, China

**Keywords:** quality control, fMRI, neuroimaging, DPABI, pipeline

## Abstract

Quality control (QC) is an important stage for functional magnetic resonance imaging (fMRI) studies. The methods for fMRI QC vary in different fMRI preprocessing pipelines. The inflating sample size and number of scanning sites for fMRI studies further add to the difficulty and working load of the QC procedure. Therefore, as a constituent part of the Demonstrating Quality Control Procedures in fMRI research topic in Frontiers, we preprocessed a well-organized open-available dataset using DPABI pipelines to illustrate the QC procedure in DPABI. Six categories of DPABI-derived reports were used to eliminate images without adequate quality. After the QC procedure, twelve participants (8.6%) were categorized as excluded and eight participants (5.8%) were categorized as uncertain. More automatic QC tools were needed in the big-data era while visually inspecting images was still indispensable now.

## 1. Introduction

Quality control (QC) is an important stage for functional magnetic resonance imaging (fMRI) studies. Images with a variety of artifacts, noticeable head motion artifacts, a low signal-to-noise ratio, inadequate slices, etc., are eliminated by researchers. Some nuisance signals such as head motion artifacts would be further regressed out and included as covariates in the following statistic. In the present study, we illustrated the fMRI quality control routine in DPABI by preprocessing a well-organized fMRI dataset.

Quality control for fMRI is becoming more challenging at this point. The challenge stems from several sources. First, to reduce the false positive rate and increase the reproducibility of an fMRI experiment, the sample size required has significantly improved over the past decade. More MRI data result in increased human power consumption in the non-automatic QC procedures such as visually screening the T1-weighted images with unacceptable motion artifacts (Backhausen et al., 2016). Second, even if the workload of researchers has been lessened by well-known preprocessing tools like fMRIPrep (Esteban et al., 2019), C-PAC (Michael et al., 2013), and DPABI (Yan et al., 2016), the optimum quality control procedures in these preprocessing pipelines still call for human involvement in the process. Several fully automatic brain MRI QC tools have been developed but the generalizability of them needs to be further validated on the independent datasets (Mortamet et al., 2009; Alfaro-Almagro et al., 2018; Bastiani et al., 2019). Third, the generalizability of findings drawn from multi-center image acquisition studies could be significantly improved. However, the variability across MR manufacturers, scanning procedures, daily scanner QC standards, and other factors may prevent researchers from applying a consistent criterion to exclude data. Therefore, a meta-data report for all the preprocessed participants would contribute to avoiding mistakes such as deficiency of time points in functional sessions or abnormal TR. In general, the present QC tools are designed to reduce the mechanically repetitive operations of users by providing and illustrating more user-friendly quality assessments. These tools may significantly alleviate the working load added by increased sample size and multi-center design, but could not replace the decision-making procedure of human beings in QC. Last but not least, the open-science data-sharing trend offers an unpretentious opportunity to reuse existing data or combine a vast number of images to carry out ambitious large-scale analyses. However, the inclusion of meta-data of samples could be various among different datasets and acquisition parameters might be unavailable for some datasets. Even worse, some flaws can be hard for users of these open datasets to identify (e.g., the flipped left-right direction, redundant images for an MR series, wrong participant sex labels, etc.). To summarize, the issues raised above demand that researchers prioritize the quality control procedure and integrate more efficient and user-friendly tools into preprocessing pipelines.

Most of the popular fMRI pipelines have their unique QC routines. The MRIQC is a pioneer specialized QC framework that incorporates a variety of techniques (Esteban et al., 2017). In recent, the main contributors of MRIQC developed another important pipeline fMRIPrep for fMRI preprocessing. The fMRIPrep would produce a series of intuitive dynamic graphs and charts to demonstrate the effectiveness of Bold-T1 image co-registration, brain surface reconstruction, spatial normalization, and the severity of head motion after fMRI preprocessing. These graphs and reports are frequently invoked by QC procedures in the other pipelines such as DPABISurf (Yan et al., 2021) and ENIGMA HALFpipe (Waller et al., 2022). For example, HALFpipe provides an interactive webpage for users to evaluate an integrated quality report derived from fMRIPrep and other tools for each participant. And DPABI also combines all the reports from a group of participants into three reports to reduce repetitive operations. As mentioned above, QC was essential for large-scale, multi-center imaging projects. Therefore, the recent large-scale projects like UKBiobank (Alfaro-Almagro et al., 2018), ABCD (Hagler et al., 2019), and ENIGMA (Waller et al., 2022) also created their own (combination of) QC methods. In addition to these specialized QC tools, imaging formatter such as DCM2NIIX (Li et al., 2016), BIDS-validator and DPABI_InputPreparer could also be used to check for the absence of imaging meta-data in QC. DPABI is a widely-used user-friendly toolbox for fMRI data processing. Both existing QC tools and in-house QC procedures have been integrated into the volume-based pipeline DPARSF, surface-based pipeline DPABISurf and specialized QC modules. The purpose of this work was to demonstrate how to QC fMRI data in DPABI. Participants with poor image quality were excluded based on a set of criteria which was thoroughly described.

## 2. Materials and methods

### 2.1. Participants

A collection of resting-state fMRI data, called fmri-open-qc-rest, was used for demonstrating the QC procedure in DPABI. The fmri-open-qc-rest dataset includes participants pooled from 7 different datasets, each with about 20 subjects (total *N* = 139). It’s a demonstrating data of the fMRI Open QC Project and the anonymous samples were selected from widely-used open-available datasets such as the functional connectome project (FCP) (Biswal et al., 2010), the autism brain imaging data exchange (ABIDE) (Di Martino et al., 2014) and the OpenNeuro resource (Markiewicz et al., 2021). The sex and age of participants were not available in the fmri-open-qc-rest dataset.

### 2.2. Surface-based MRI preprocessing

Both a volume-based pipeline (DPARSF) and a surface-based pipeline (DPABISurf) in DPABI were used to preprocess the MRI data. Surface-based methods are increasingly common in the most recent studies and are superior to volume-based methods in terms of structure localization, spatial smoothing, and reproducibility (Coalson et al., 2018). However, the surface-based methods were time-consuming and omitted the analysis of subcortical and cerebellar areas. The volume-based approaches would be appropriate for conducting whole-brain analysis, preprocessing large datasets, etc. Additionally, the DPARSF pipelines reorient/QC module offered a user-friendly graphical user interface for visually assessing the image quality before the remaining laborious stages (e.g., structure segmentation).

In specific, surface-based preprocessing was performed by DPABISurf (Yan et al., 2021), a surface-based fMRI data analysis toolbox evolved from DPABI/DPARSF. DPABISurf used docker technology to wrap the whole computing environment for fMRIPrep (Esteban et al., 2019), FreeSurfer (Fischl, 2012), ANTs (Tustison et al., 2014), FSL (Jenkinson et al., 2012), AFNI (Cox, 1996), SPM (Ashburner, 2012), GNU Parallel (Tange, 2011), PALM (Winkler et al., 2014), MATLAB (The MathWorks Inc., Natick, MA, USA), Docker^1^ and DPABI (Yan et al., 2016), etc. The pipelines mentioned above have their own preprocessing and QC procedures and an elaborate comparison among these pipelines could be found in the ENIGMA HALFpipe references (Waller et al., 2022). The resting-state functional images and T1-weighted images were preprocessed by the following steps. (1) Checking the BIDS JSON-format image meta-data; (2) intensity non-uniformity correction and skull-stripping; (3) tissue segmentation of cerebrospinal fluid (CSF), white matter (WM), and gray matter (GM); (4) brain surface reconstruction; (5) deleting initial 10 time points; (6) boundary-based registration of BOLD and T1 images; (7) BOLD image spatial normalization to fsaverage5 space; (8) head-motion, WM, and CSF signal and linear trend nuisance regression; (9) bandpass filtering (0.01–0.1 Hz); (10) spatial smoothing [full-width at half-maximum (FWHM) of 6 mm]. Detailed preprocessing procedures can be found in our previous research (Chen and Yan, 2021).

Of note, slice-timing corrections were not conducted because there were errors in the slice-timing information of some participants. Normally, DPABISurf/DPARSF would read the slice-timing information from DICOM header files (if the input images were in DICOM format) and metadata files in the BIDS format or the DPABI format (if the input images were in NIFTI format). As the demonstrating data in the fmri-open-qc-rest dataset were in NIFTI format, the slice-timing correction procedures would use the related metadata in the BIDS schema. The related information such as acquisition time for each slice and the scanning sequence (e.g., interleave or sequence while scanning different slices in a volume) were recorded in separated JSON files in the BIDS data-structure and could not be extracted from the NIFTI images themselves. In the fmri-open-qc-rest dataset, slice-timing-related information of some participants was missing or incorrect. The exact details were provided in see Section “3.2. Issues in MRI meta-data.” Therefore, we skipped the slice-timing correction while this procedure might be necessary for the images with a relatively long repetition time (Sladky et al., 2011) (e.g., TR = 2.5 for most of the participants in the dataset).

### 2.3. Volume-based MRI preprocessing

Volume-based data preprocessing in our study was carried out using the Data Processing Assistant for resting-state fMRI (DPARSF) (Yan and Zang, 2010), which was based on SPM (Friston et al., 1994) and had been integrated into Data Processing and Analysis of Brain Imaging (DPABI) (Yan et al., 2016). The first 10 time-points of the fMRI series were discarded. The head motion was corrected by a six-parameter (rigid body) linear transformation with a two-pass procedure (Yan et al., 2013). Reorient/QC was a module in DPARSF pipeline for both adjusting the orientation of the images and visually checking the image quality of each T1-weighted or BOLD image. We rated each image by a 5-point scale. The 5-point rating scales provided semiquantitative scores for the results of the visually evaluation in reorient/QC module. More points equaled better images. The derived reports would record both the rating scores and the comments for images. After the whole Reorient/QC procedures were finished, a QC-score-threshold of 3 was set in the following dialog box. The images with extremely bad quality were not be involved in the further preprocessing procedure to avoid contaminating other samples in the certain procedures (e.g., creating a group template). After coregistering the structural and functional images and unified segmentation (Ashburner and Friston, 2005) on T1 image, spatial normalization to MNI-152 space [a coordinate system created by Montreal Neurological Institute (Fonov et al., 2009)] was performed using the Diffeomorphic Anatomical Registration Through Exponentiated Lie algebra (DARTEL) tool (Goto et al., 2013). The Friston 24-parameter model (Friston et al., 1996) was applied to regress out head motion effects. White matter signal, cerebrospinal fluid signal and linear trends were regressed out from each voxel’s time course. Finally, all images were filtered by temporal bandpass filtering (0.01–0.1 Hz) to reduce the effect of low-frequency drift and high-frequency physiological noise.

### 2.4. Quality control procedure

**FIGURE 1:**
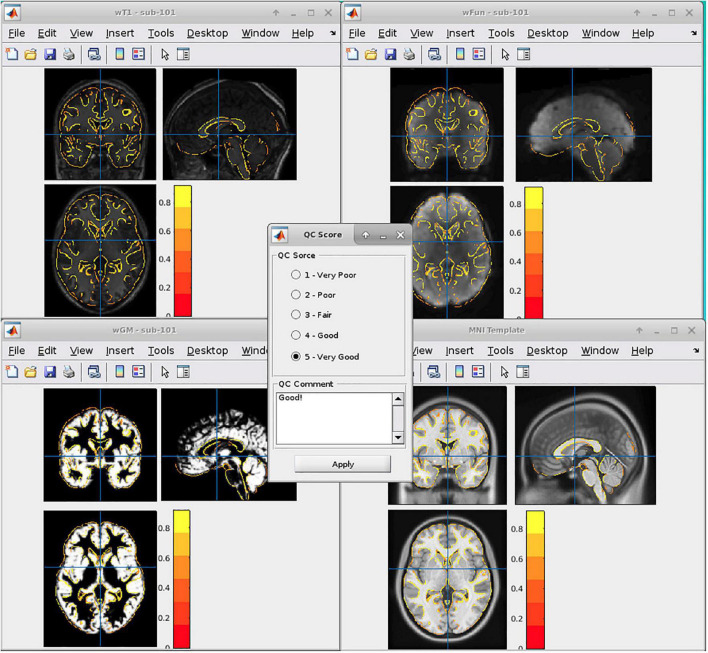
Graphic user interfaces of the spatial normalization quality control (QC) tools in DPABI.

In general, we adopted six DPABI-derived reports to exclude participants with insufficient quality. The detailed criteria according to the reports were listed in Table 1. The QC procedures were integrated into two pipelines with graphic user interfaces (GUI) for the volume-based methods and surface-based methods. A detailed introduction to these modules could be found in the related course at http://rfmri.org/Course. An intuitive exclusive tool for checking spatial normalization quality in the volume-based preprocessing was displayed in Figure 1. The detailed criteria for eliminating samples derived from these reports were listed in Table 1.

**TABLE 1:** Resting state functional magnetic resonance imaging (fMRI) quality control (QC) criteria: Exclude a subject if.

Index	Criteria	Derived from
A1	Low brain coverage (quantitative and qualitative)	DPARSF, QC report
A2	Severe signal loses in temporal lobe (qualitative)	DPARSF, QC report
A3	Head-motion related artifacts (qualitative)	DPARSF, QC report
A4	Other MRI artifacts (qualitative)	DPARSF, QC report
A5	Flipped/Uncertain scan direction (qualitative)	DPARSF, QC report
A6	Anomalous structural occupancy or abnormity (qualitative)	DPARSF, QC report
B1	Maximum head-motion exceeding 3 mm or 3 degree (quantitative)	DPARSF/DPABISurf, Realign parameters
B2	Averaged framewise displacements exceeding 0.2 (quantitative)	DPARSF/DPABISurf, Realign parameters
C1	Bad BOLD-T1 co-registration (qualitative)	DPABISurf, QC_EPItoT1 report
C2	Head-motion related artifacts (qualitative)	DPABISurf, QC_EPItoT1 report
C3	Other MRI artifacts (qualitative)	DPABISurf, QC_EPItoT1 report
C4	Flipped/Uncertain scan direction (qualitative)	DPABISurf, QC_EPItoT1 report
D1	Bad brain surface reconstruction (qualitative)	DPABISurf, QC_SurfaceReconstruction report
D2	Bad skull stripping (qualitative)	DPABISurf, QC_SurfaceReconstruction report
E1	Bad spatial normalization (qualitative)	DPABISurf, QC_T1toMNI report
E2	Head-motion-related artifacts (qualitative)	DPABISurf, QC_T1toMNI report
E3	Other MRI artifacts (qualitative)	DPABISurf, QC_T1toMNI report
E4	Low signal-to-noise ratio (qualitative)	DPABISurf, QC_T1toMNI report
E5	Anomalous structural occupancy or abnormity (qualitative)	DPABISurf, QC_T1toMNI report
F1	Abnormal TR, number of volumes, etc., (quantitative)	DPARSF/DPABISurf, Meta-data report

“Other MRI artifacts” indicate a variety of visually recognizable MRI artifacts, including susceptibility artifacts, wraparound artifacts, coil-related artifacts, chemical artifacts, etc.

A. The QC rating scores derived from the Reorient/QC module in the DPARSF pipeline. The Reorient/QC module is a GUI designed for visually checking and manually orientation-adjusting the raw T1-weighted and functional images. The QC scores for each subject were given by the user according to the imaging quality. Subjects with structural or functional image QC scores below 3 would not be included in further preprocessing.
B. The head-motion reports from DPABISurf/DPARSF pipeline. There were two reports about the head-motion of participants. The first one was a brief report for excluding participants according to several commonly-used rules (e.g., maximum rigid displacement or rotation exceeding 3 mm or 3 degrees). The second one was a detailed head-motion report spreadsheet recording the head-motion in different directions and the framewise displacements (FD) would be used as another threshold of mean head-motion (Jenkinson et al., 2002). We set a mean FD-Jenkinson head-motion threshold to 0.2.
C. The dynamic graph for checking co-registration between structural images and functional images of each participant derived from DPABISurf pipeline. Bad BOLD-T1 co-registration, MRI artifacts and flipped image direction can be distinguished from this report.
D. The dynamic graph for checking brain surface reconstruction for each participant derived from DPABISurf pipeline. Bad brain surface reconstruction can be distinguished from this report. Of note, bad skull stripping may lead to inaccurate surface reconstruction and structural metrics estimation and can be recognized in this report.
E. The dynamic graph for checking spatial normalization from individual space to standard (MNI) space of each participant derived from DPABISurf pipeline. Bad spatial normalization, MRI artifacts, low signal-to-noise ratio, anomalous structural occupancy or abnormity can be distinguished from this report. The three graphical reports (e.g., co-registration, surface reconstruction and spatial normalization) of every participant were summarized into three HTML page in the derived QC folder in the DPABI working directory.
F. The meta-data report spreadsheet (TRInfo.tsv) of images generated by DPARSF or DPABISurf. Abnormal meta-data records such as a smaller number of volumes, atypical TR and strange voxel sizes can be distinguished from this report. This report was considered a unique QC resource in DPABI because the mistakenly included images and incomplete images could be easily discriminated using the meta-data reports.

### 2.5. Sex difference with/without quality control

To preliminarily illustrate the effect of quality control in statistical analysis, we conducted two-sample *t*-tests to show the sex differences in some common fMRI metrics. Of note, a comprehensive evaluation of the QC-effect in group-level analysis (e.g., taking into account the site-effect and the reduced sample size after eliminating samples) would be a larger and separate topic. Importantly, the sex labels of the participants were not provided by the organizers of fmri-open-qc-rest dataset and we used a T1-weighted image-based classifier to predict the sex of each participant (Lu et al., 2022). Considering the sex classifier achieved about 95% accuracy, we supposed that the estimated classifier values would be close to the ground truth. Sex differences were tested in both the images with QC and the images without any QC. For the statistics without QC, thirteen estimated male participants and seven estimated female participants were excluded. The fMRI metrics included regional homogeneity (ReHo), (fractional) amplitude of low-frequency fluctuations (fALFF/ALFF) and degree centrality (DC). The sites and the mean FD-Jenkinson scores were included as covariates. The statistical maps of the two-sample *t*-tests were corrected for family-wise error rate (FWER) using Gaussian random field (GRF) correction. The vertex-wise threshold was 0.001 and the cluster-wise threshold for GRF correction was 0.017 (0.05/3, 3 for Bonferroni correction of two hemispheres and one subcortical area).

## 3. Results

### 3.1. Quality control summary

In sum, 12 participants were excluded after quality control in DPABI and 8 participants might be further excluded on a stricter standard, accounting for 8.6 and 5.8% of the whole fmri-open-qc-rest sample (please see a detailed excluding list with subject ID in Supplementary Table 1). The detailed QC criteria were described in the following sections. The orders of these sections were determined by the frequency of being triggered and the importance of the excluding criterion in each section (e.g., from high to low).

### 3.2. Issues in MRI meta-data

There were several potential issues in the meta-data of images that were identified before preprocessing.

Firstly, the functional images in site-2 and site-5 could not pass the BIDS metadata validation procedure in DPABI. The bids-validation tools reported that “slice-timing values contain invalid value as it is greater than the repetition time” for five participants (e.g., from sub-501 to sub-504, sub-509). Therefore, the five participants with the specific slice-timing errors were labeled as “uncertain,” as we suspected the acquisition sequences were thoroughly distorted. In addition, some of the participants did not have any slice-timing information in the BIDS schema. As we skipped the slice-timing correction in preprocessing, these participants were not excluded from the present study.

Secondly, the number of volumes (time points) was not consistent in site-1 and site-6. It may be acceptable for site-6 as we anticipate that site-6 were constructed by multiple sub-site. But the two participants (e.g., sub-114 and sub-115) with fewer volumes compared with the others in site-1 may suggest data loss in practice. We did not label these suspicious samples as “uncertain” or “excluded” as we did not know the actual scanning protocols for these participants. However, we still raised this frequently occurring issue (inconsistent number of volumes for the images with the same scanning protocol) to inform the beginner of MRI data processing.

Thirdly, sub-605 had two runs of the BOLD series in the raw data while the others only had one run in each session. No additional information was available to help determine which run was more appropriate for further processing. We arbitrarily used the latter one and did not exclude this participant. Because in the practice, the additional run of an MRI series was probably due to the unsatisfying quality of the previous run of the same series (e.g., head-motion exceeding the criteria).

### 3.3. Head-motion related artifacts

**FIGURE 2:**
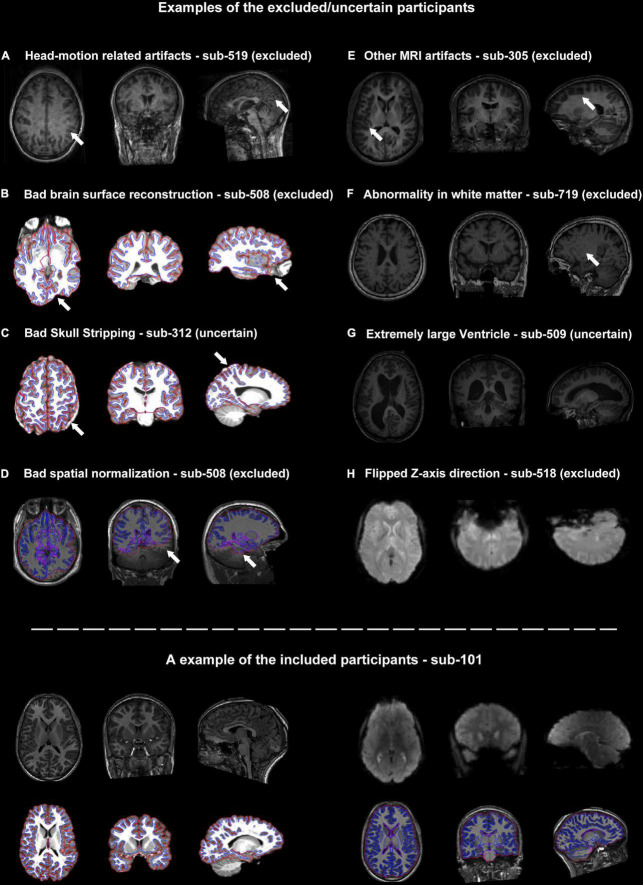
Representative examples of quality control (QC) items for which subjects were categorized as excluded or uncertain. **(A–H)** Examples of images with inadequate quality. The suspicious areas were highlighted using white arrows. The lower panel of the graph showed an example of the included participants.

The head-motion induced artifacts were the most frequently reported issue in the QC procedure. Seven out of twenty “uncertain” or “excluded” participants were potentially excluded due to unacceptable head-motion. Some of them were visually identified and the others were identified by the head-motion report generated by DPARSF/DPABISurf (Figure 2A). Of note, the criteria related to the head-motion should be determined according to the research topic (Nebel et al., 2022).

### 3.4. Bad brain surface reconstruction

The core procedure of the surface-based methods was brain surface reconstruction. The surface reconstruction could fail due to a variety of quality problems (e.g., low brain coverage of field of view, low signal-to-noise ratio, abnormal brain structure and imaging artifacts, Figure 2B). In addition, the low quality of skull stripping may also hamper accurate surface reconstruction (Figure 2C).

### 3.5. Bad spatial normalization

There were two structural images of the participants that failed to achieve satisfying spatial normalization (Figure 2D). Spatial normalization (and related structural segmentation) could fail due to the low quality of images and local minimum in optimization induced by certain random seeds under extremely rare circumstances. Spatial normalization could be substantially improved by the reorientation procedure (e.g., manually rigid translation and rotation before spatial normalization) in DPARSF.

### 3.6. Other MRI artifacts

Besides head-motion, there are many MRI artifacts that could affect the image quality, including magnetic susceptibility artifacts, wraparound artifacts, coil-related artifacts, chemical artifacts and et al. (e.g., the T1-weighted images of sub-305 were blurred by unknow MRI artifacts, Figure 2E).

### 3.7. Abnormal brain structures

It’s very challenging for neuroscientists to distinguish abnormal brain structures from normal anatomy or tiny MRI artifacts (Figures 2F, G). For example, sub-509 was labeled as uncertain because of the large ventricle. The QC classifiers of the UKBiobank would also take “Bad registration: Structurally atypical: Big Ventricles” as a problem situation. However, large ventricles might be common in the aged population and may not relate to disorders. Therefore, the eliminating criteria could be changeable according to the aim of the studies.

### 3.8. Flipped Z-axis direction

The functional MRIs of two subjects (sub-518 and sub-519) were flipped along the z-axis (Figure 2H). These results underlined the importance of visually checking the images. Flipped images along z-axis (up-down) could be further reversed and are less destructive, but images flipping along the x-axis (left-right) would be harder to recognize and would significantly affect brain symmetry research.

### 3.9. Sex differences with/without quality control

**FIGURE 3:**
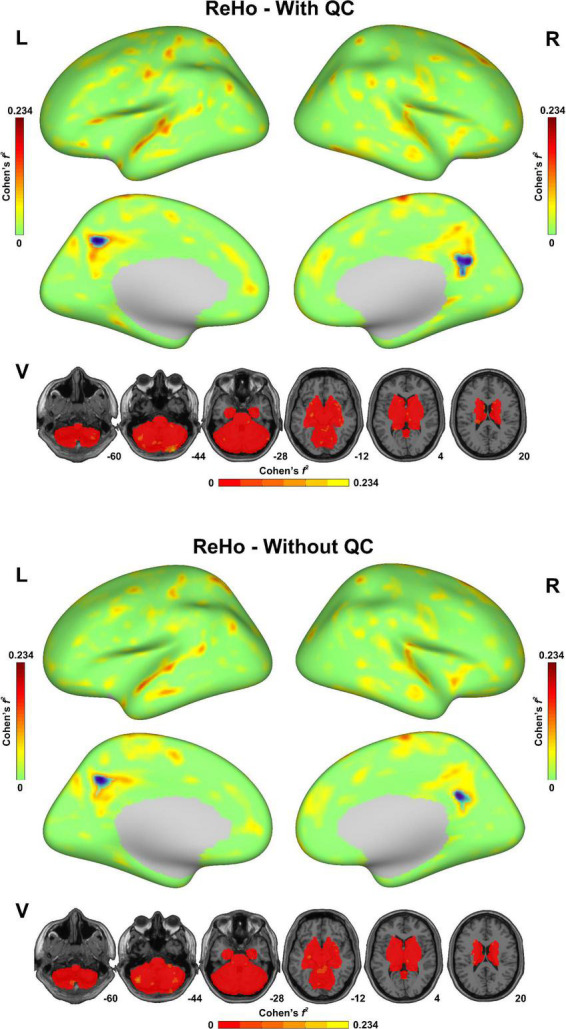
Sex difference of regional homogeneity (ReHo) in the fmri-open-qc-rest dataset with/without quality control. The effect size (Cohen’s *f*^2^) derived from the two-sample *t*-tests between males and females were displayed. The brain areas showed significantly lower ReHo in the male group than in the female group were highlighted in blue. L indicated the left hemisphere. R indicated the right hemisphere. V indicated the subcortical area.

As shown in Figure 3, both of the statistical maps of ReHo sex differences (with/without QC) showed significantly decreased spontaneous activity strength in the posterior cingulate cortex in the male group, which was consistent with the pre-existing literature (Chen et al., 2018). However, the maximal effect size values (Cohen’s *f*^2^) with QC (0.234 in the left hemisphere, 0.173 in the right hemisphere and 0.161 in the subcortical area) were higher than that without QC (0.221 in the left hemisphere, 0.152 in the right hemisphere and 0.153 in the subcortical area). Similarly, the maximal effect size values in the sex difference statistical maps of DC, fALFF, and ALFF with QC were higher than that without QC (Supplementary Figures 1–3).

## 4. Discussion

In the present study, a well-organized open-available MRI dataset was quality controlled by DPABI pipelines both in volume space and surface space. Twenty (14.4%) participants were categorized as excluded or uncertain. The reasons for these participants to be excluded could be summarized into eight categories: MRI meta-data issues, head-motion related artifacts, bad brain surface reconstruction, bad spatial normalization, other MRI artifacts, abnormal brain structures, and flipped images. In general, we believed that the QC procedure in DPABI could effectively improve the validity of the following analysis.

As mentioned in the description of fMRI Open QC Project, there is no single correct way to do QC. The criteria (thresholds) should be adjusted according to the population and the aim of the study. For example, head-motion related artifacts are still the most prevalent reason for excluding participants. Three types of criteria for controlling head-motion effect were used in the present study: (1) visual screening, (2) thresholding maximum head-motion, and (3) thresholding mean FD-Jenkinson. For studies whose research population is children or brain disorder patients, setting a strict threshold may dramatically reduce the available samples which is not acceptable for some longitudinal studies. While for studies in which head-motion artifacts must be minimized, some time-consuming but effective algorithms such as ICA-AROMA (Pruim et al., 2015) could be used to further remove head-motion effects. Another example is that participants with extremely large ventricles might be excluded from a group of children, but might be kept in a group of aged participants. In addition, all the QC criteria should be taken into account to determine the imaging quality of a participant. For example, the quality of skull stripping is low for both sub-312 and sub-315. But sub-312 was categorized as “uncertain” while sub-315 was categorized as “excluded” due to the additional uncertain structural occupancy and artifact on the parietal lobe. In addition, some of the QC procedures in DPABI were not conducted in the present study. For example, ICA-AROMA is an outstanding algorithm to control head-motion related artifacts based on independent component analysis (ICA). As this algorithm is extremely time-consuming, it is an optional method in DPABISurf but is not conducted in default, while some other pipelines would include ICA-based nuisance regressions using a modified ICA-AROMA algorithm (Waller et al., 2022). Moreover, a detailed list of exclusion criteria and excluded subject IDs in the studies based on public datasets would save time for other researchers and improve the reproducibility of the findings.

Eliminating participants with bad image quality is a critical procedure to improve the quality of research. In a broader sense, the quality control in fMRI research should also include the daily scanner QC using water phantom, contraindications inspection (e.g., metal braces) while recruiting participants, correct patient positioning, head-motion suppression using sponge mat or optimized coil, avoiding meta-data loss at image archive platforms, checking critical meta-data before preprocessing, carefully eliminating participants using QC reports generated by preprocessing pipelines, rigorous coding and statistic, etc. The acquisition protocols also interact with the QC procedure. For example, the multiband acquisition could improve the temporal resolution but decrease the signal-to-noise ratio (SNR) (Smith et al., 2013). Therefore, the SNR should be included as an important criterion in studies using multiband protocols. Discussing all these procedures is out of the scope of the present study, but the steps mentioned above would also influence participant-eliminating.

Therefore, more automatic QC tools are critical. For example, the sex of participants could be mistakenly recorded, and this mistake is hard to recognize. Recently, a T1-weighted image-based classifier trained using more than 85,000 MRI samples from more than 217 sites/scanners achieved 95% accuracy in a sex classification task on the independent datasets. This sex classifier could be an *Ex post* check procedure for sex labels.^2^ As mentioned in the results 3.8 section, flipped images along the x-axis (left-right) would be a very subtle situation that is not easy to distinguish. The oil capsule marks for labeling left or right are not available for every dataset and the tricks [e.g., brain torque (Toga and Thompson, 2003)] for visual checks may not work for every participant. Fortunately, an efficient tool built in the AFNI fMRI processing procedure that can automatically distinguish the flipped images has been developed (Glen et al., 2020). Besides the specialized QC modules in DPABI, the input preparer module and the data organization checking module could also help avoid including incomplete images. And a new harmonization module in DPABI containing comprehensive multi-center imaging harmonizing methods would be available soon. In addition, as mentioned in the introduction, the design philosophy of DPABI was to minimize the repetitive and non-standardized human involvement in fMRI preprocessing, but the decision-making part of human involvement is still unavoidable. The UKBiobank imaging team has developed an automated machine learning based QC tool which performed excellently on the UKBiobank dataset. However, the UKBiobank’s scanning protocols are uniform across all of the scanning sites, which might result in overfitting and poor generalizability. The generalizability of this promising tool needs to be further validated on a variety of datasets.

In summary, the QC procedures for fMRI in DPABI are illustrated by preprocessing a well-organized open dataset. A set of reports derived from DPABI pipelines could be utilized for excluding images with bad quality. More automatic QC tools are needed in the big-data era while visually inspecting images is still indispensable.

## Data availability statement

The datasets presented in this study can be found in online repositories. The names of the repository/repositories and accession number(s) can be found below: https://osf.io/qaesm/wiki/home/; https://github.com/Chaogan-Yan/PaperScripts/blob/master/Lu_2023_fMRIQC.

## Ethics statement

The studies involving human participants were reviewed and approved by Institute of Psychology. The patients/participants provided their written informed consent to participate in this study.

## Author contributions

C-GY designed the overall experiment and the QC tools. BL carried out the QC procedure. Both authors contributed to the article, wrote the manuscript, and approved the submitted version.

## Footnotes

1
https://docker.com

2
http://brainimagenet.org

## References

[B1] Alfaro-Almagro F. Jenkinson M. Bangerter N. K. Andersson J. L. R. Griffanti L. Douaud G. (2018). Image processing and quality control for the first 10,000 brain imaging datasets from UK Biobank. *Neuroimage* 166 400–424. 10.1016/j.neuroimage.2017.10.034 29079522 PMC5770339

[B2] Ashburner J. (2012). SPM: A history. *Neuroimage* 62 791–800. 10.1016/j.neuroimage.2011.10.025 22023741 PMC3480642

[B3] Ashburner J. Friston K. J. (2005). Unified segmentation. *Neuroimage* 26 839–851. 10.1016/j.neuroimage.2005.02.018 15955494

[B4] Backhausen L. L. Herting M. M. Buse J. Roessner V. Smolka M. N. Vetter N. C. (2016). Quality control of structural mri images applied using freesurfer-a hands-on workflow to rate motion artifacts. *Front. Neurosci.* 10:558. 10.3389/fnins.2016.00558 27999528 PMC5138230

[B5] Bastiani M. Cottaar M. Fitzgibbon S. P. Suri S. Alfaro-Almagro F. Sotiropoulos S. N. (2019). Automated quality control for within and between studies diffusion MRI data using a non-parametric framework for movement and distortion correction. *Neuroimage* 184 801–812. 10.1016/j.neuroimage.2018.09.073 30267859 PMC6264528

[B6] Biswal B. B. Mennes M. Zuo X. N. Gohel S. Kelly C. Smith S. M. (2010). Toward discovery science of human brain function. *Proc. Natl. Acad. Sci. U. S. A.* 107 4734–4739. 10.1073/pnas.0911855107 20176931 PMC2842060

[B7] Chen X. Yan C. G. (2021). Hypostability in the default mode network and hyperstability in the frontoparietal control network of dynamic functional architecture during rumination. *Neuroimage* 241:118427. 10.1016/j.neuroimage.2021.118427 34311069

[B8] Chen X. Lu B. Yan C. G. (2018). Reproducibility of R-fMRI metrics on the impact of different strategies for multiple comparison correction and sample sizes. *Hum. Brain Mapp.* 39 300–318. 10.1002/hbm.23843 29024299 PMC6866539

[B9] Coalson T. S. Van Essen D. C. Glasser M. F. (2018). The impact of traditional neuroimaging methods on the spatial localization of cortical areas. *Proc. Natl. Acad. Sci. U. S. A.* 115 E6356–E6365. 10.1073/pnas.1801582115 29925602 PMC6142239

[B10] Cox R. W. (1996). AFNI: Software for analysis and visualization of functional magnetic resonance neuroimages. *Comput. Biomed. Res.* 29 162–173. 10.1006/cbmr.1996.0014 8812068

[B11] Di Martino A. Yan C. G. Li Q. Denio E. Castellanos F. X. Alaerts K. (2014). The autism brain imaging data exchange: Towards a large-scale evaluation of the intrinsic brain architecture in autism. *Mol. Psychiatry* 19 659–667. 10.1038/mp.2013.78 23774715 PMC4162310

[B12] Esteban O. Birman D. Schaer M. Koyejo O. O. Poldrack R. A. Gorgolewski K. J. (2017). MRIQC: Advancing the automatic prediction of image quality in MRI from unseen sites. *PLoS One* 12:e0184661. 10.1371/journal.pone.0184661 28945803 PMC5612458

[B13] Esteban O. Markiewicz C. J. Blair R. W. Moodie C. A. Isik A. I. Erramuzpe A. (2019). fMRIPrep: A robust preprocessing pipeline for functional MRI. *Nat. Med.* 16 111–116. 10.1038/s41592-018-0235-4 30532080 PMC6319393

[B14] Fischl B. (2012). FreeSurfer. *Neuroimage* 62 774–781. 10.1016/j.neuroimage.2012.01.021 22248573 PMC3685476

[B15] Fonov V. S. Evans A. C. Mckinstry R. C. Almli C. R. Collins D. L. (2009). Unbiased nonlinear average age-appropriate brain templates from birth to adulthood. *Neuroimage* 47. 10.1016/S1053-8119(09)70884-5

[B16] Friston K. J. Holmes A. P. Worsley K. J. Poline J. P. Frith C. D. Frackowiak R. S. (1994). Statistical parametric maps in functional imaging: A general linear approach. *Hum. Brain Mapp.* 2 189–210. 10.1002/hbm.460020402

[B17] Friston K. J. Williams S. Howard R. Frackowiak R. S. Turner R. (1996). Movement-related effects in fMRI time-series. *Magn. Reson. Med.* 35 346–355. 10.1002/mrm.1910350312 8699946

[B18] Glen D. R. Taylor P. A. Buchsbaum B. R. Cox R. W. Reynolds R. C. (2020). Beware (surprisingly common) left-right flips in your mri data: An efficient and robust method to check mri dataset consistency using AFNI. *Front. Neuroinform.* 14:18. 10.3389/fninf.2020.00018 32528270 PMC7263312

[B19] Goto M. Abe O. Aoki S. Hayashi N. Miyati T. Takao H. (2013). Diffeomorphic anatomical registration through exponentiated lie algebra provides reduced effect of scanner for cortex volumetry with atlas-based method in healthy subjects. *Neuroradiology* 55 869–875. 10.1007/s00234-013-1193-2 23619702

[B20] Hagler D. J. Jr. Hatton S. Cornejo M. D. Makowski C. Fair D. A. Dick A. S. (2019). Image processing and analysis methods for the adolescent brain cognitive development study. *Neuroimage* 202:116091. 10.1016/j.neuroimage.2019.116091 PMC6981278 31415884

[B21] Jenkinson M. Bannister P. Brady M. Smith S. (2002). Improved optimization for the robust and accurate linear registration and motion correction of brain images. *Neuroimage* 17 825–841. 10.1006/nimg.2002.1132 12377157

[B22] Jenkinson M. Beckmann C. F. Behrens T. E. Woolrich M. W. Smith S. M. (2012). Fsl. *Neuroimage* 62 782–790. 10.1016/j.neuroimage.2011.09.015 21979382

[B23] Li X. Morgan P. S. Ashburner J. Smith J. Rorden C. (2016). The first step for neuroimaging data analysis: DICOM to NIfTI conversion. *J. Neurosci. Methods* 264 47–56. 10.1016/j.jneumeth.2016.03.001 26945974

[B24] Lu B. Li H. X. Chang Z. K. Li L. Chen N. X. Zhu Z. C. (2022). A practical Alzheimer’s disease classifier via brain imaging-based deep learning on 85,721 samples. *J. Big Data* 9:101. 10.1186/s40537-022-00650-y

[B25] Markiewicz C. J. Gorgolewski K. J. Feingold F. Blair R. Halchenko Y. O. Miller E. (2021). The OpenNeuro resource for sharing of neuroscience data. *Elife* 10:e71774. 10.7554/eLife.71774 34658334 PMC8550750

[B26] Michael M. Francisco C. Adriana D. M. Clare K. Maarten M. Stanley C. (2013). Towards automated analysis of connectomes: The configurable pipeline for the analysis of connectomes (C-PAC). *Front. Neuroinform.* 7. 10.3389/conf.fninf.2013.09.00042

[B27] Mortamet B. Bernstein M. A. Jack C. R. Jr. Gunter J. L. Ward C. Britson P. J. (2009). Automatic quality assessment in structural brain magnetic resonance imaging. *Magn. Reson. Med.* 62 365–372. 10.1002/mrm.21992 19526493 PMC2780021

[B28] Nebel M. B. Lidstone D. E. Wang L. Benkeser D. Mostofsky S. H. Risk B. B. (2022). Accounting for motion in resting-state fMRI: What part of the spectrum are we characterizing in autism spectrum disorder? *Neuroimage* 257:119296. 10.1016/j.neuroimage.2022.119296 35561944 PMC9233079

[B29] Pruim R. H. R. Mennes M. van Rooij D. Llera A. Buitelaar J. K. Beckmann C. F. (2015). ICA-AROMA: A robust ICA-based strategy for removing motion artifacts from fMRI data. *Neuroimage* 112 267–277. 10.1016/j.neuroimage.2015.02.064 25770991

[B30] Sladky R. Friston K. J. Trostl J. Cunnington R. Moser E. Windischberger C. (2011). Slice-timing effects and their correction in functional MRI. *Neuroimage* 58 588–594. 10.1016/j.neuroimage.2011.06.078 21757015 PMC3167249

[B31] Smith S. M. Beckmann C. F. Andersson J. Auerbach E. J. Bijsterbosch J. Douaud G. (2013). Resting-state fMRI in the human connectome project. *Neuroimage* 80 144–168. 10.1016/j.neuroimage.2013.05.039 23702415 PMC3720828

[B32] Tange O. (2011). Gnu parallel-the command-line power tool. *USENIX Mag.* 36 42–47.

[B33] Toga A. W. Thompson P. M. (2003). Mapping brain asymmetry. *Nat. Rev. Neurosci.* 4 37–48. 10.1038/nrn1009 12511860

[B34] Tustison N. J. Cook P. A. Klein A. Song G. Das S. R. Duda J. T. (2014). Large-scale evaluation of ANTs and FreeSurfer cortical thickness measurements. *Neuroimage* 99 166–179. 10.1016/j.neuroimage.2014.05.044 24879923

[B35] Waller L. Erk S. Pozzi E. Toenders Y. J. Haswell C. C. Buttner M. (2022). ENIGMA HALFpipe: Interactive, reproducible, and efficient analysis for resting-state and task-based fMRI data. *Hum. Brain Mapp.* 43 2727–2742. 10.1002/hbm.25829 35305030 PMC9120555

[B36] Winkler A. M. Ridgway G. R. Webster M. A. Smith S. M. Nichols T. E. (2014). Permutation inference for the general linear model. *Neuroimage* 92 381–397. 10.1016/j.neuroimage.2014.01.060 24530839 PMC4010955

[B37] Yan C. G. Zang Y. F. (2010). DPARSF: A MATLAB toolbox for “pipeline” data analysis of resting-state fMRI. *Front. Syst. Neurosci.* 4:13. 10.3389/fnsys.2010.00013 20577591 PMC2889691

[B38] Yan C. G. Cheung B. Kelly C. Colcombe S. Craddock R. C. Di M. A. (2013). A comprehensive assessment of regional variation in the impact of head micromovements on functional connectomics. *Neuroimage* 76 183–201. 10.1016/j.neuroimage.2013.03.004 23499792 PMC3896129

[B39] Yan C. G. Wang X. D. Lu B. (2021). DPABISurf: Data processing & analysis for brain imaging on surface. *Sci. Bull.* 66 2453–2455. 10.1016/j.scib.2021.09.016 36654202

[B40] Yan C. G. Wang X. D. Zuo X. N. Zang Y. F. (2016). DPABI: Data processing & analysis for (resting-state) brain imaging. *Neuroinformatics* 14 339–351. 10.1007/s12021-016-9299-4 27075850

