# Supplementary material for: Demonstrating quality control procedures for fMRI in DPABI
Source: Front Neurosci. 2023 Feb 21;17:1069639. doi: 10.3389/fnins.2023.1069639 (PMC9989208; doi:10.3389/fnins.2023.1069639)
Supplement: Supplementary file 1 [file Data_Sheet_1.docx]

Supplementary Material

TABLE S1. Excluded/uncertain subjects in resting state data-sites

| SUBJ ID | EXCLUDE | UNCERTAIN | COMMENT (failed QC criteria, etc.) |
| --- | --- | --- | --- |
| sub-102 | X |  | **B1,** Maximum head-motion exceeding 3 mm or 3 degree |
| sub-114 |  | X | **F1,** Lower number of volumes in BOLD image |
| sub-115 |  | X | **F1,** Lower number of volumes in BOLD image |
| sub-203 | X |  | **E1,** Bad spatial normalization on cerebellum |
| sub-305 | X |  | **A4,** Severe MRI artifacts in T1 image |
| sub-307 | X |  | **B2,** Averaged framewise displacements exceeding 0.2 |
| sub-312 |  | X | **D2,** Bad skull stripping |
| sub-315 | X |  | **A6,** Uncertain structural occupancy; **D2,** Bad skull stripping; **E3,** Uncertain artifact on parietal lobe |
| sub-501 |  | X | **F1,** Slice-timing values contain invalid value as it is greater than repetition time (TR) |
| sub-502 |  | X | **F1,** Slice-timing values contain invalid value as it is greater than repetition time (TR) |
| sub-503 |  | X | **F1,** Slice-timing values contain invalid value as it is greater than repetition time (TR) |
| sub-504 |  | X | **A3,** Severe head-motion; **F1,** Slice-timing values contain invalid value as it is greater than repetition time (TR) |
| sub-508 | X |  | **D1,** Bad brain surface reconstruction; **E1,** Bad spatial normalization on cerebellum |
| sub-509 |  | X | **A6,** Extremely large ventricle; **F1,** Slice-timing values contain invalid value as it is greater than repetition time (TR) |
| sub-518 | X |  | **A5,** Flipped the z-axis direction |
| sub-519 | X |  | **A3,** Severe head-motion in T1 image; **A5,** flipped the z-axis direction; **B1,** Maximum head-motion exceeding 3 mm or 3 degree; **B2,** Averaged framewise displacements exceeding 0.2; **D1,** Bad brain surface reconstruction |
| sub-620 | X |  | **B1,** Maximum head-motion exceeding 3 mm or 3 degree |
| sub-706 | X |  | **B2,** Averaged framewise displacements exceeding 0.2 |
| sub-714 | X |  | **B1**, Maximum head-motion exceeding 3 mm or 3 degree; **B2,** Averaged framewise displacements exceeding 0.2 |
| sub-719 | X |  | **E5,** Anomalous structural abnormity in white matter |


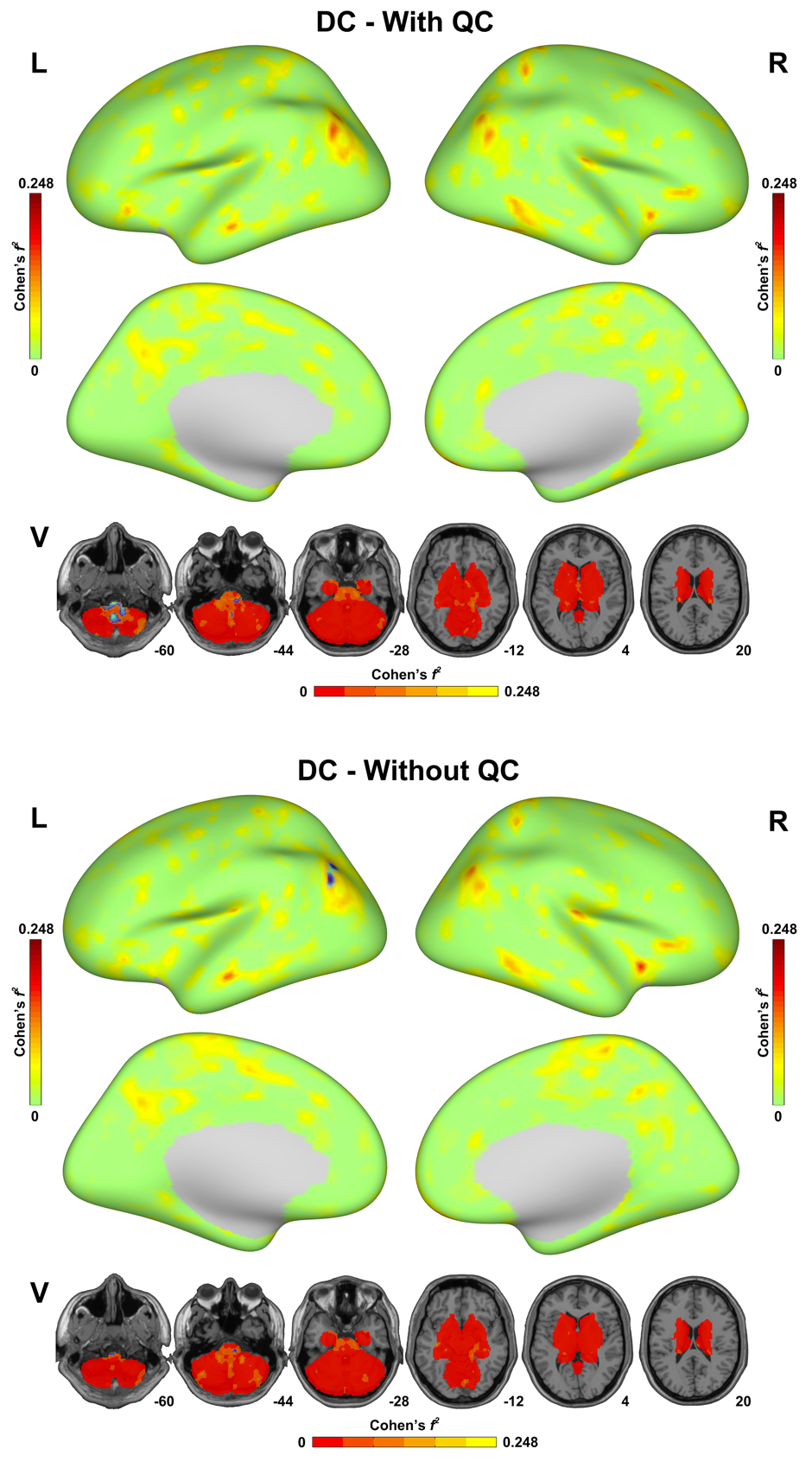


FIGURE S1. Sex difference of degree centrality (DC) in the fmri-open-qc-rest dataset with/without quality control. The effect size (Cohen’s *f^2^*) derived from the two-sample *t*-tests between males and females was displayed. The brain areas showed significantly lower DC in the male group than in the female group were highlighted in blue. L indicated the left hemisphere. R indicated the right hemisphere. V indicated the subcortical area.


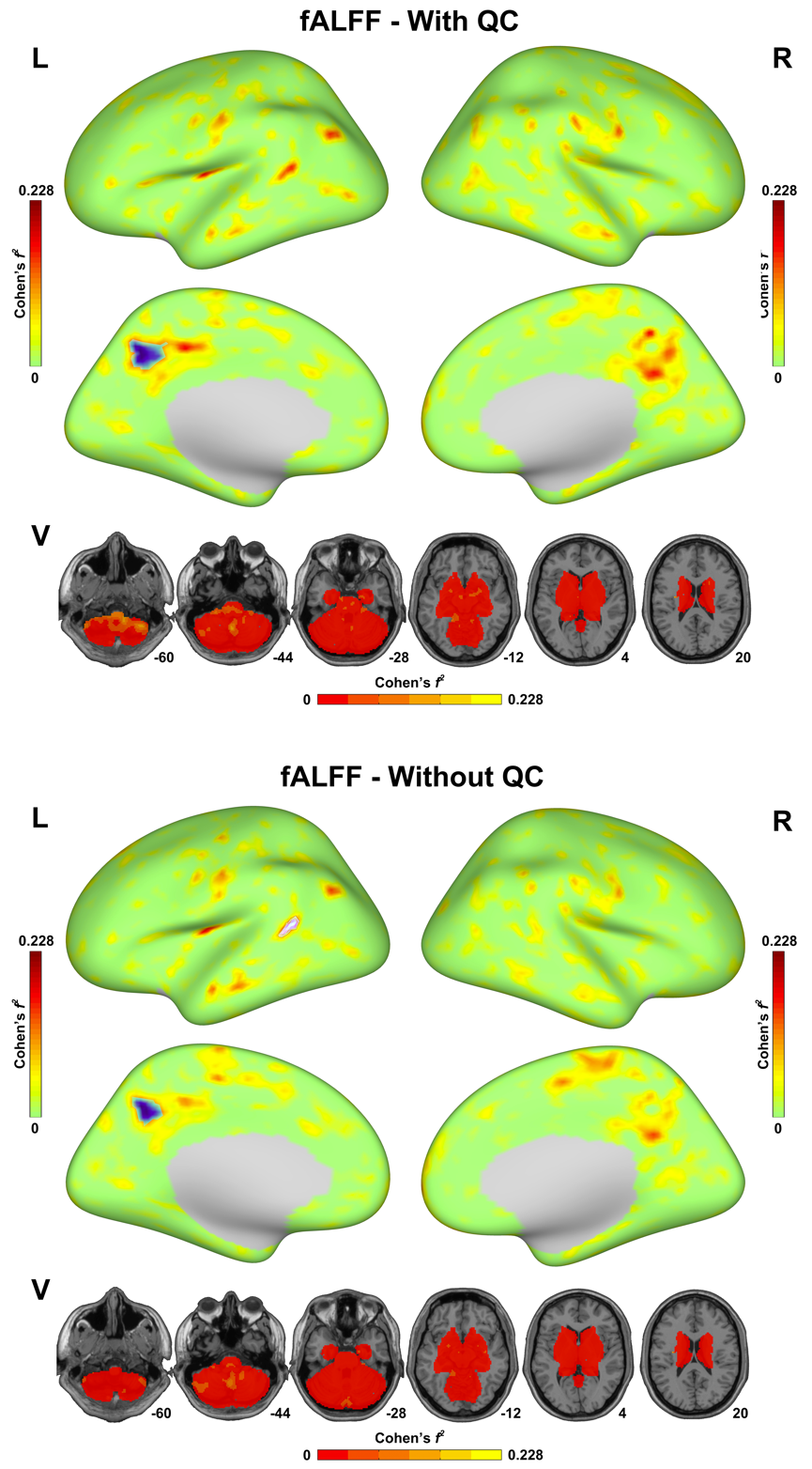


FIGURE S2. Sex difference of fractional amplitude of low-frequency fluctuations (fALFF) in the fmri-open-qc-rest dataset with/without quality control. The effect size (Cohen’s *f^2^*) derived from the two-sample *t*-tests between males and females was displayed. The brain areas showed significantly lower fALFF in the male group than in the female group were highlighted in blue. The brain areas showed significantly higher fALFF in the male group than in the female group were highlighted in pink. L indicated the left hemisphere. R indicated the right hemisphere. V indicated the subcortical area.


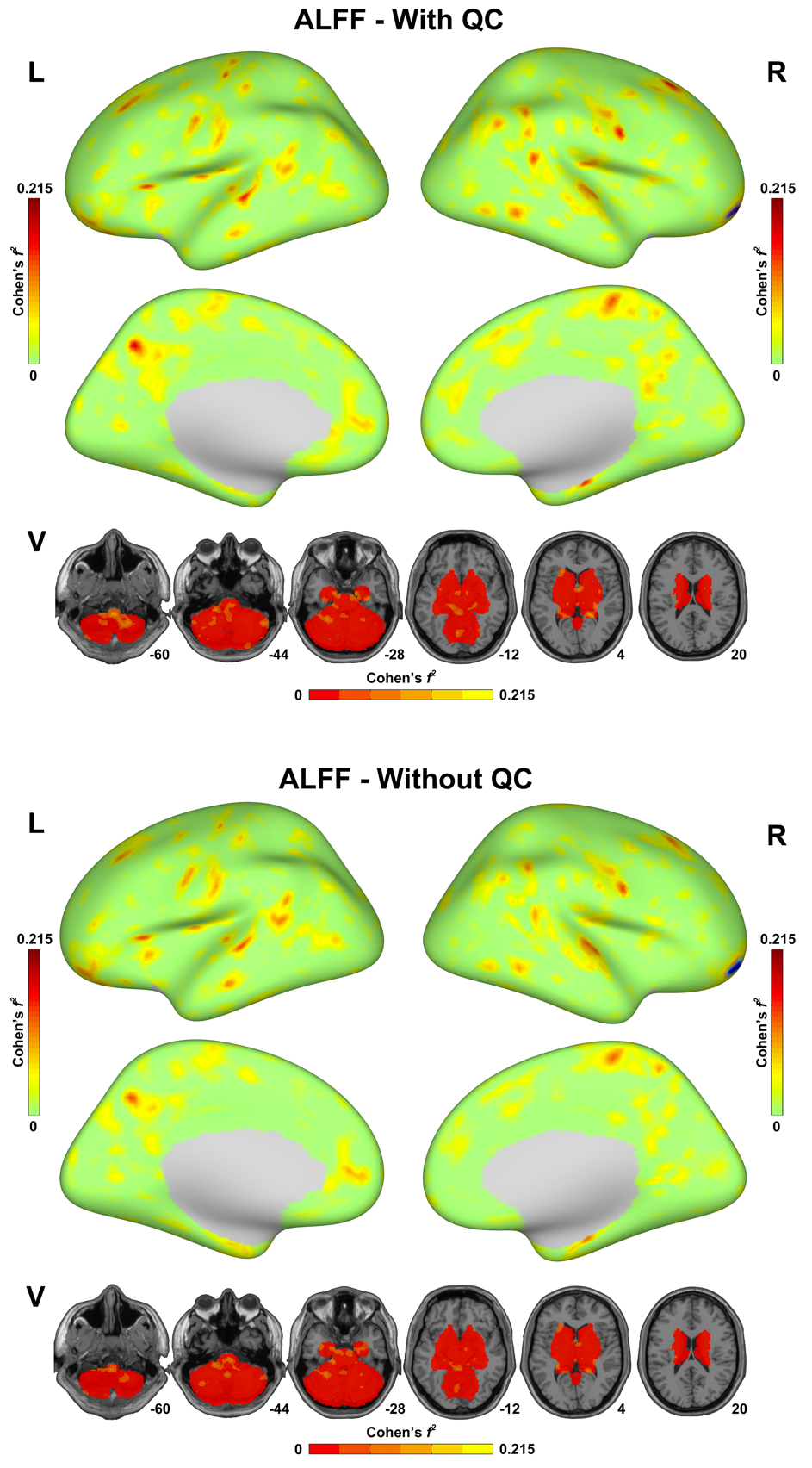


FIGURE S3. Sex difference of amplitude of low-frequency fluctuations (ALFF) in the fmri-open-qc-rest dataset with/without quality control. The effect size (Cohen’s *f^2^*) derived from the two-sample *t*-tests between males and females was displayed. The brain areas showed significantly lower ALFF in the male group than in the female group were highlighted in blue. L indicated the left hemisphere. R indicated the right hemisphere. V indicated the subcortical area.
